# Uric Acid as a Redox Switch in Gout: Linking Xanthine Oxidoreductase-Derived ROS, NLRP3 Inflammasome Activation and Emerging Ferroptotic Mechanisms

**DOI:** 10.3390/antiox15080993

**Published:** 2026-08-11

**Authors:** Petar-Preslav Petrov, Delyan Dimitrov, Darina Barbutska, Zlatina Nikolova, Nikoleta Dimitrova

**Affiliations:** 1 Department of Anatomy, Histology and Embryology, Faculty of Medicine, Medical University of Plovdiv, 4002 Plovdiv, Bulgaria; 2 Faculty of Medicine, Medical University of Plovdiv, 4002 Plovdiv, Bulgaria; 3 Department of Midwifery Care, Faculty of Public Health, Medical University of Plovdiv, 4002 Plovdiv, Bulgaria; 4 Department of Occupational Diseases Including Clinical Allergology Activities, University Multiprofile Hospital for Active Treatment “St. George”, 4002 Plovdiv, Bulgaria; 5 Department of Occupational Diseases, Clinical Allergology and Toxicology, Faculty of Medicine, Medical University of Plovdiv, 4002 Plovdiv, Bulgaria

**Keywords:** gout, uric acid, redox signaling, xanthine oxidoreductase, NLRP3 inflammasome, neutrophil extracellular traps, lipid peroxidation, ferroptosis

## Abstract

Gout is a crystal-induced inflammatory arthritis driven by hyperuricemia and monosodium urate (MSU) crystal deposition, yet urate burden alone does not explain why only a subset of hyperuricemic individuals develops clinical disease, why acute flares are usually self-limited, or why gout clusters with renal and cardiometabolic comorbidity. This structured narrative review uses a gout-specific redox-switch framework, defined as a context-dependent shift in uric acid biology according to concentration, compartment, crystallization state, xanthine oxidoreductase (XOR) activity, inflammatory priming, and disease stage rather than as a binary molecular event. We integrate evidence on XOR-derived reactive oxygen species (ROS), mitochondrial stress, NLRP3 inflammasome signaling, neutrophil oxidative responses, neutrophil extracellular traps (NETs), lipid peroxidation, and potential ferroptosis-related mechanisms. Evidence is classified into five categories: established, mechanistically supported, human-associative, conceptual, and emerging/unvalidated. The available human data support lipid-peroxidation and ferroptosis-associated molecular signatures, but do not yet establish ferroptotic cell death as a driver of gout. Therapeutic implications are therefore framed conservatively: urate-lowering therapy remains foundational, whereas redox-directed approaches require pathway specificity, disease-stage definition, and biomarker validation. The redox-switch concept is proposed as an organizing framework for mechanistic and translational research, not as a validated clinical algorithm.

## 1. Introduction

Gout is crystal-induced inflammatory arthritis caused by monosodium urate (MSU) crystal deposition in joints and periarticular tissues. Hyperuricemia is a necessary biochemical substrate for crystal formation, but it is not sufficient to account for the full biological and clinical spectrum of the disease. Many individuals with elevated serum urate remain asymptomatic for years, whereas others develop recurrent flares, intercritical inflammation, chronic synovitis, tophus formation, erosive joint damage, nephrolithiasis, chronic kidney disease, and cardiometabolic comorbidity. Gout should therefore be interpreted as an interaction among urate burden, crystal formation, innate immune activation, metabolic stress, and tissue-specific redox regulation [1,2,3,4,5].

The global burden of hyperuricemia and gout continues to rise, although these conditions are not epidemiologically equivalent. The Global Burden of Disease (GBD) 2021 gout analysis estimated 55.8 million prevalent gout cases in 2020 and projected approximately 95.8 million prevalent cases by 2050 [1]. A subsequent GBD 2023 analysis reported a 158.2% increase in global incident gout cases from 1990 to 2023 and a shift of incident disease toward older age groups [6]. In parallel, a 2026 global modelling study estimated that approximately 305 million women and 500 million men were living with hyperuricemia in 2023 [7]. The much larger population with hyperuricemia than with clinically manifest gout reinforces the central premise that serum urate concentration alone does not determine whether crystal deposition becomes symptomatic inflammatory disease. Renal dysfunction further modifies this burden and contributes to substantial gout-related disability [8]. Epidemiological reviews likewise emphasize rising prevalence, undertreatment, and a dense burden of renal, metabolic, and cardiovascular comorbidity [4,5]. This trajectory makes mechanistic refinement clinically important: understanding why hyperuricemia becomes inflammatory gout may reveal biomarkers and therapeutic windows that are not captured by serum urate concentration alone.

Current management appropriately emphasizes sustained urate lowering, flare prophylaxis, acute flare control, and treat-to-target serum urate strategies [9,10]. These interventions remain the clinical foundation because lowering urate prevents new crystal formation and promotes dissolution of existing deposits. Nevertheless, serum urate values do not directly report local crystal burden, inflammasome activation, neutrophil behavior, oxidative damage, or resolution biology. The same urate concentration may therefore coexist with markedly different inflammatory states.

MSU crystals are canonical danger-associated molecular patterns in gout. They interact with macrophages, synoviocytes, neutrophils, and other resident or recruited cells, providing activation signals for the NLRP3 inflammasome and promoting caspase-1-dependent maturation of interleukin-1β (IL-1β) [11,12,13,14,15,16]. This pathway transformed gout from a predominantly physicochemical crystal disorder into a prototype of sterile inflammation. NLRP3 activation, however, is embedded in a broader environment shaped by lysosomal injury, ionic fluxes, mitochondrial dysfunction, XOR activity, NADPH oxidases, antioxidant reserve, neutrophil oxidative burst, and lipid oxidation.

In this review, the term “redox switch” denotes a context-dependent change in the biological consequences of uric acid rather than a discrete molecular on/off mechanism. The relevant determinants include urate concentration, extracellular versus intracellular location, solubility and crystallization, XOR activity, antioxidant reserve, mitochondrial function, innate immune priming, and disease stage. This definition is introduced at the outset to avoid implying that uric acid has a single fixed antioxidant or prooxidant identity.

The central conceptual contribution of this review is to integrate urate biology, XOR-derived ROS, NLRP3 activation, neutrophil-driven amplification and resolution, and lipid-peroxidation pathways within a stage-aware gout model. The framework accommodates the antioxidant contribution of extracellular urate while recognizing that intracellular, enzymatic, crystal-associated, and inflammatory contexts can favor oxidative signaling and tissue injury [17,18,19,20,21,22].

The framework is anchored in foundational observations that uric acid can function as an endogenous danger signal released during tissue injury, that MSU-driven inflammation requires IL-1 receptor/MyD88 signaling in experimental systems, and that urate crystals can activate immune cells through receptor-independent membrane interactions and Syk-related signaling [23,24,25]. These findings support a model in which crystal burden is necessary, but the magnitude and fate of inflammation depend on the local immunometabolic environment.

Existing reviews have addressed gout epidemiology, NLRP3 biology, neutrophil extracellular traps, and ferroptosis-related hypotheses [26,27,28,29]. The present review does not attempt to duplicate those syntheses. Its novelty lies in organizing these pathways according to redox context, evidence maturity, and disease stage, while explicitly distinguishing measurable lipid peroxidation from ferroptosis-specific regulated cell death.

This positioning also avoids the clinically misleading conclusion that nonspecific antioxidant supplementation is broadly beneficial in gout. Contemporary redox biology distinguishes physiological redox signaling from oxidative molecular damage and cautions against treating “oxidative stress” as a generic therapeutic indication [30,31,32,33,34,35,36]. Accordingly, the therapeutic discussion prioritizes defined pathways, experimentally characterized compounds, translational limitations, and biomarker-guided validation.

**Figure 1:**
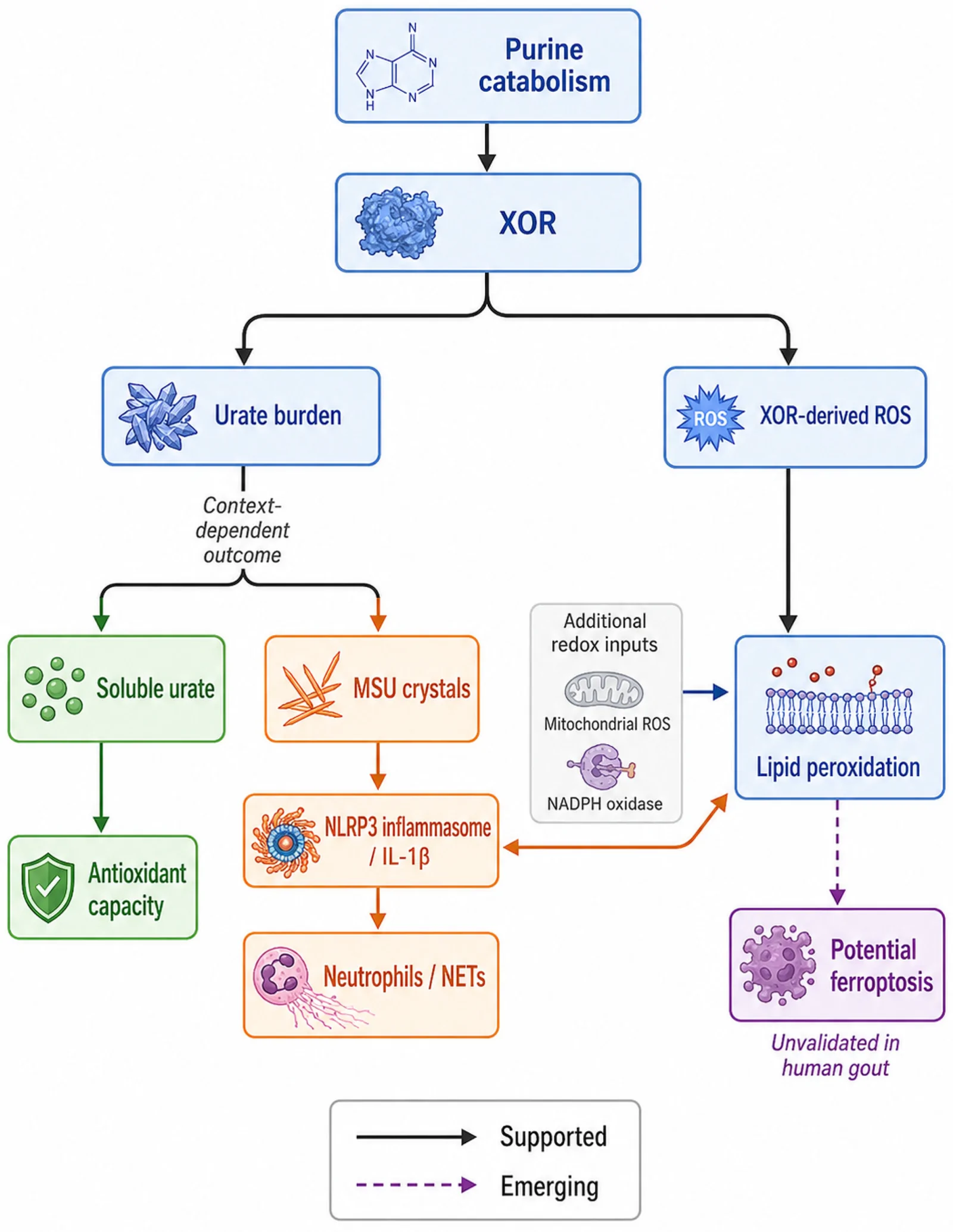
The uric acid redox-switch framework in gout. Purine catabolism links xanthine oxidoreductase (XOR) activity to urate generation and reactive oxygen species production. Soluble extracellular urate may contribute to antioxidant capacity in a context-dependent manner, whereas persistent urate burden promotes monosodium urate (MSU) crystallization, NLRP3 inflammasome activation, interleukin-1β release, and neutrophil/NET responses. XOR-derived ROS, mitochondrial ROS, and NADPH oxidase-dependent oxidative burst provide additional redox inputs that may promote inflammasome signaling and lipid peroxidation. The dashed arrow from lipid peroxidation to potential ferroptosis denotes an emerging relationship that has not been validated as regulated ferroptotic cell death in human gout. Solid arrows indicate clinically established or mechanistically supported relationships. The figure provides a simplified visual synthesis of the principal pathways discussed in this review.

The proposed model is summarized in Figure 1.

## 2. Literature Search Approach and Scope of the Review

### 2.1. Review Design and Database Search

This article was designed as a structured narrative review rather than as a systematic review or meta-analysis. The final database search was conducted on 1 August 2026 in Scopus and the Web of Science Core Collection. Searches were restricted to publications from 2020 through 2026 and were organized into three database-adapted thematic blocks: (i) urate, xanthine oxidoreductase (XOR), reactive oxygen species (ROS), and NLRP3 inflammasome signaling; (ii) neutrophils, neutrophil extracellular traps (NETs), lipid peroxidation, and ferroptosis; and (iii) Nrf2, redox-modulating interventions, biomarkers, and epidemiology.

The following Scopus searches were performed:

Scopus Search 1—urate/XOR/ROS/NLRP3:

TITLE-ABS-KEY((gout OR “gouty arthritis” OR hyperuricemia OR “monosodium urate” OR “MSU crystal*”) AND (“uric acid” OR urate OR “xanthine oxidoreductase” OR “xanthine oxidase”) AND (“reactive oxygen species” OR ROS OR “oxidative stress” OR redox OR NLRP3 OR inflammasome OR “IL-1 beta” OR mitochondrial)) AND PUBYEAR > 2019 AND PUBYEAR < 2027

This search retrieved 1667 records.

Scopus Search 2—neutrophils/NETs/lipid peroxidation/ferroptosis:

TITLE-ABS-KEY((gout OR “gouty arthritis” OR “monosodium urate” OR “MSU crystal*”) AND (neutrophil* OR NETosis OR “neutrophil extracellular trap*” OR “lipid peroxidation” OR ferroptosis OR GPX4 OR ACSL4 OR ALOX15 OR “lipid ROS” OR “iron metabolism”)) AND PUBYEAR > 2019 AND PUBYEAR < 2027

This search retrieved 611 records.

Scopus Search 3—Nrf2/interventions/biomarkers/epidemiology:

TITLE-ABS-KEY((gout OR “gouty arthritis” OR hyperuricemia OR “monosodium urate”) AND (Nrf2 OR KEAP1 OR antioxidant* OR polyphenol* OR phytochemical* OR biomarker* OR proteomic* OR metabolomic* OR epidemiolog* OR prevalence OR incidence OR “global burden”)) AND PUBYEAR > 2019 AND PUBYEAR < 2027

This search retrieved 7691 records.

The following Web of Science Core Collection searches were performed:

Web of Science Search 1—urate/XOR/ROS/NLRP3:

TS=((gout OR “gouty arthritis” OR hyperuricemia OR “monosodium urate” OR “MSU crystal*”) AND (“uric acid” OR urate OR “xanthine oxidoreductase” OR “xanthine oxidase”) AND (“reactive oxygen species” OR ROS OR “oxidative stress” OR redox OR NLRP3 OR inflammasome OR “IL-1 beta” OR mitochondrial)) AND PY=(2020–2026)

This search retrieved 1805 records.

Web of Science Search 2—neutrophils/NETs/lipid peroxidation/ferroptosis:

TS=((gout OR “gouty arthritis” OR “monosodium urate” OR “MSU crystal*”) AND (neutrophil* OR NETosis OR “neutrophil extracellular trap*” OR “lipid peroxidation” OR ferroptosis OR GPX4 OR ACSL4 OR ALOX15 OR “lipid ROS” OR “iron metabolism”)) AND PY=(2020–2026)

This search retrieved 356 records.

Web of Science Search 3—Nrf2/interventions/biomarkers/epidemiology:

TS=((gout OR “gouty arthritis” OR hyperuricemia OR “monosodium urate”) AND (Nrf2 OR KEAP1 OR antioxidant* OR polyphenol* OR phytochemical* OR biomarker* OR proteomic* OR metabolomic* OR epidemiolog* OR prevalence OR incidence OR “global burden”)) AND PY=(2020–2026)

This search retrieved 5119 records.

Across the six search sets, 17,249 records were exported: 9969 from Scopus and 7280 from Web of Science. Records were exported in RIS format. Where database export limits required batching, the Web of Science records were exported in separate batches and subsequently combined before deduplication.

PubMed/MEDLINE was used for targeted verification of candidate records, bibliographic metadata, recently published original studies, and indispensable foundational publications. Verification searches addressed gout epidemiology and global burden, uric acid redox biology, XOR-derived ROS, MSU/NLRP3 signaling, soluble-urate priming, NET-mediated amplification and resolution, lipid peroxidation and ferroptosis, Nrf2-directed interventions, and candidate biomarkers. PubMed/MEDLINE records were not added to the Scopus/Web of Science record-count denominator unless they were already represented in the exported search sets.

### 2.2. Record Management, Eligibility, and Evidence Appraisal

Deduplication was performed using normalized digital object identifiers (DOIs) and, when a DOI was unavailable, normalized article titles. This procedure yielded 10,500 unique records and removed 6749 duplicate occurrences. One record carrying 2027 issue metadata was retained only for bibliographic verification and was not treated as falling within the prespecified 2020–2026 publication window.

The unique records were computationally organized and thematically prioritized to support candidate identification. This procedure was used as an organizational aid and did not constitute a formal systematic-review screening workflow. Candidate studies incorporated into the narrative synthesis were subsequently evaluated using bibliographic metadata, abstracts, and full-text sources where available.

English-language original human clinical and translational studies were given the highest priority when they directly informed the proposed redox-switch framework. Original animal and in vitro studies were included when they provided mechanistic evidence that could not be obtained from human studies. Clinical guidelines and high-quality reviews were used for clinical context, conceptual comparison, and backward or forward citation chaining. Publications predating 2020 were retained when they established indispensable mechanisms that had not been superseded, including MSU-induced NLRP3 activation, uric acid danger signaling, XOR-dependent inflammasome regulation, and aggregated NET-mediated resolution.

Records were excluded from detailed consideration when they addressed hyperuricemia without direct mechanistic or translational relevance to gout, consisted solely of in silico target prediction without experimental validation, or duplicated evidence already represented by a stronger or more directly relevant primary source. Descriptive natural-product studies without pathway-level evidence or adequate experimental characterization were not used to support affirmative therapeutic conclusions.

Evidence was appraised according to experimental context: human clinical or translational evidence, animal-model evidence, in vitro mechanistic evidence, and secondary synthesis. Candidate publications were assessed for direct relevance to gout, study design, biological compartment, disease stage, exposure conditions, pathway specificity, and whether the findings supported association, prediction, mechanism, or intervention.

For emerging pathways, particularly ferroptosis and the ROS-related effects of XOR inhibition, molecular association was distinguished from functional causation and from validated clinical relevance. Pathway enrichment, altered expression of a single protein, or the presence of a nonspecific oxidative-damage marker was not considered sufficient evidence of regulated ferroptotic cell death. Similarly, experimental reduction of ROS was not interpreted as evidence of a clinically meaningful urate-independent effect of XOR inhibition.

Study selection was based on conceptual and translational relevance rather than on a prespecified systematic-review protocol. The review was not registered, no formal risk-of-bias instrument was applied, and no quantitative meta-analysis was performed. These limitations are acknowledged explicitly because the search and appraisal procedures were intended to support a structured narrative synthesis rather than to produce an exhaustive systematic evidence estimate.

### 2.3. Positioning Against Adjacent Reviews

The literature contains focused reviews on gout epidemiology, clinical management, MSU crystal biology, NLRP3 activation, NET-mediated inflammation and resolution, and ferroptosis-related pathways [1,2,4,9,10,13,14,15,16,26,27,28,29,37,38,39,40]. The present article addresses a different integrative question: how can uric acid redox biology, XOR-derived ROS, inflammasome activation, neutrophil responses, lipid oxidation, evidence maturity, and disease stage be combined within a translationally useful framework for gout?

The review is therefore organized around mechanisms, evidence boundaries, and translational questions rather than around lists of antioxidant compounds. Natural products and redox-modulating interventions are discussed only after the disease model has been established and are evaluated according to experimental model, exposure, pathway dependence, mechanistic endpoint, and available human evidence. This structure reduces conceptual redundancy and avoids presenting the literature as a descriptive nutraceutical catalogue.

### 2.4. Terminological Note on the “Redox Switch”

The term “redox switch” is used as conceptual shorthand for the context-dependent biological consequences of uric acid. It does not imply a single molecular switch, a universally fixed direction of effect, or a validated clinical classification. Its purpose is organizational: to distinguish urate burden from redox reactivity, crystallization, and crystal-triggered immune activation, while accounting for biological compartment, XOR activity, antioxidant reserve, inflammatory priming, and disease stage.

## 3. Uric Acid as an Antioxidant–Prooxidant Redox Switch

Uric acid is the final product of purine degradation in humans. Because humans lack functional uricase, circulating urate concentrations are higher than in most mammals. In extracellular fluids, urate contributes to antioxidant capacity and can scavenge selected reactive oxygen and nitrogen species [17,18]. This antioxidant contribution explains why urate cannot be classified as uniformly harmful and why concentration alone is an incomplete descriptor of its biology.

The oxidant–antioxidant paradox arises because the biological consequences of urate change with compartment and chemical context. Plasma urate can contribute to antioxidant defense, whereas intracellular uptake, reduced nitric oxide bioavailability, metal-dependent reactions, inflammatory priming, and crystallization can favor prooxidant or proinflammatory effects [17,18,19,41]. In gout, the decisive transition is not simply from a low to a high serum concentration, but from soluble urate burden to supersaturation, crystal formation, and a permissive inflammatory microenvironment.

Recent original studies illustrate why this duality should not be reduced to a single direction of effect. At physiological concentrations, soluble uric acid was identified as a reversible non-competitive inhibitor of CD38, limiting NAD+ degradation and reducing crude-LPS- and MSU-induced inflammation in mouse models [42]. Population data also associate a more favorable oxidative-balance score with lower serum urate and a lower prevalence of hyperuricemia [43], whereas a 2026 case–control study of 100 patients with gout and 100 controls reported higher circulating MDA and hs-CRP together with lower total antioxidant capacity and glutathione [44]. These observational findings support systemic redox imbalance but are not joint-specific and cannot establish causality. Together, the evidence reinforces a compartment-, concentration-, and disease-context-dependent interpretation.

Relevant determinants include concentration, pH, temperature, sodium availability, extracellular versus intracellular localization, renal and intestinal handling, XOR activity, mitochondrial state, antioxidant reserve, endothelial function, and coexisting metabolic inflammation. The same measured serum urate may therefore coexist with different crystal loads and different redox-inflammatory states.

Upstream urate handling is heterogeneous. URAT1/SLC22A12-mediated reabsorption, ABCG2-mediated renal and extra-renal export, gout classification, and polygenic control shape the urate burden on which redox-inflammatory mechanisms operate [45,46,47,48]. These transport and genetic determinants are not redox pathways themselves, but they define exposure duration and tissue supersaturation.

The practical implication is that serum urate remains an essential clinical target but cannot serve as a surrogate for local oxidative injury, inflammasome activity, NET biology, or ferroptosis-specific cell death. A useful framework must therefore distinguish urate burden, crystallization, redox reactivity, and immune activation rather than collapsing them into a single continuum.

## 4. Xanthine Oxidoreductase: Linking Urate Production to ROS Generation

Xanthine oxidoreductase (XOR) catalyzes the oxidation of hypoxanthine to xanthine and xanthine to uric acid. Depending on enzyme form, substrate availability, and tissue context, XOR can also generate superoxide and hydrogen peroxide. XOR is therefore a dual-output node linking purine catabolism to both urate generation and redox signaling.

In clinical gout, allopurinol and febuxostat are prescribed to reduce urate production. Their proven disease-modifying benefit is mediated by sustained serum urate reduction, prevention of new crystal formation, and dissolution of existing deposits [9,10]. Although inhibition of XOR can also alter ROS production, the independent contribution of ROS reduction to clinical gout outcomes has not been established in humans.

Mechanistic evidence is strongest in experimental macrophage systems. Ives and colleagues showed that XOR activity regulated NLRP3-dependent caspase-1 activation and IL-1β secretion, with xanthine oxidase-derived ROS functioning upstream of cytokine release [49]. Subsequent experimental and pharmacological studies reported that febuxostat or other XOR-directed strategies can alter inflammasome assembly, purine salvage, cellular bioenergetics, and redox signaling [50,51,52,53]. These data demonstrate biological plausibility but do not prove that a urate-independent XOR-ROS mechanism materially improves human gout.

Human treatment data remain confounded because XOR inhibitors lower urate and alter multiple downstream processes simultaneously. In two treat-to-target urate-lowering cohorts, serum and leukocyte proteomics identified treatment-emergent changes in complement and inflammatory networks after 48 weeks of allopurinol- or febuxostat-based therapy [54]. The study is translationally informative, but it did not isolate ROS-specific effects from urate lowering, changing crystal burden, or declining flare activity.

A definitive human test would require parallel measurement of serum urate, oxypurines, systemic or tissue XOR activity, validated oxidative-damage markers, crystal burden, flare outcomes, and—where feasible—synovial or tophus tissue. Mediation analyses or experimental designs that achieve comparable urate lowering with different effects on XOR-derived ROS would be needed to determine whether ROS modulation adds clinical benefit independently of urate reduction.

Tissue-resolved human evidence is also limited. Beyond treatment-associated serum and leukocyte proteomics [54], the structured search did not identify sufficiently resolved gout studies comparing XDH/XOR transcript abundance, protein expression, enzymatic activity, or oxidase-to-dehydrogenase state across synovium, synovial fluid cells, tophus tissue, circulating leukocytes, liver, kidney, and vascular compartments. This distinction matters because circulating or hepatic measurements may not represent the redox environment of the gouty joint. Comparative transcriptomic, proteomic, spatial, and activity-based profiling across disease stages is therefore a specific research priority.

Accordingly, this review treats XOR-derived ROS as a strong mechanistic link and a priority research target, but not as a validated urate-independent therapeutic mechanism in human gout. The major redox sources and pathway-level consequences are summarized in Table 1.

## 5. MSU Crystals, Mitochondrial ROS, and NLRP3 Inflammasome Activation

The NLRP3 inflammasome is one of the best-established inflammatory pathways in gout. MSU crystals activate NLRP3-dependent caspase-1 signaling and promote maturation and secretion of IL-1β and IL-18 [11,12]. This mechanism supports the clinical efficacy of IL-1 pathway blockade in selected difficult cases and explains why NLRP3 remains an active pharmacological target [15,26,27,29,55,56].

Inflammasome biology is commonly described as a priming-plus-activation process [57,58]. Priming increases NLRP3 and pro-IL-1β expression, often through NF-κB-dependent signaling, whereas activation is promoted by particulate uptake, ionic fluxes, lysosomal injury, mitochondrial dysfunction, or related stress signals. In gout, MSU crystals provide a potent activation signal, while metabolic inflammation, tissue injury, microbial products, or soluble mediators can alter the priming threshold.

Particulate-induced phagosomal destabilization and NF-κB-dependent licensing provide mechanistic context for MSU-induced inflammation [59,60]. Soluble urate can alter cytokine responses and prime primary human cells, but the effect depends on cell type and co-stimulation [61]. In primary human PBMCs and THP-1 cells, two urate-dissolving methods produced broadly similar cytokine effects and no MSU crystals were detected; primary PBMCs nevertheless required additional stimulation for a stronger response [62]. This supports a modulatory or priming role rather than equivalence with crystal-triggered activation.

Mitochondrial ROS can amplify this network, but they should not be presented as the sole upstream cause of NLRP3 assembly. MSU exposure can produce lysosomal perturbation, potassium efflux, thioredoxin-interacting protein signaling, mitochondrial injury, oxidized mitochondrial DNA, and metabolic reprogramming [63,64]. In primary human PBMCs from patients with gout, MSU alone produced only a small increase in IL-1β and limited transcriptomic change, whereas palmitate co-stimulation amplified cytokine release [65]. A 2025 human genetic and translational study further linked a gout-risk allele regulating IRF5 expression to enhanced IL-1β production in response to palmitate and MSU crystals [66]. Separately, a study using gout-patient-derived macrophages and a rat model found that ATP and MSU acted synergistically through P2X7R [67]. These observations support a multi-signal model in which host genetics, metabolic priming, and purinergic context shape the inflammatory threshold.

The frequently cited soluble-urate study by Braga and colleagues requires careful qualification [68]. Murine bone-marrow-derived macrophages were exposed to 180 μM soluble uric acid together with 10 ng/mL lipopolysaccharide; IL-1β release was NLRP3- and MyD88-dependent and lower than that induced by canonical particulate stimuli. Thus, the experiment used a primed murine system rather than soluble urate alone. Moreover, the same concentration did not induce IL-1β release in human monocyte-derived macrophages in the reported experiments, although anti-inflammatory cytokine patterns changed. The concentration is within the broad human serum range, but serum concentration does not establish intracellular exposure or exclude microcrystal formation in tissues.

More recent murine macrophage studies extend, but do not remove, these limitations. Inhibition of the urate exporter ABCG2 increased the soluble-urate-associated IL-1β response in LPS-primed, MSU-stimulated J774.1 cells, linking intracellular urate handling to inflammasome output [69]. RNA sequencing in the same cell lineage showed that soluble-urate pretreatment enhanced pro-inflammatory and M1-associated transcription and genes related to MSU phagocytosis, again in an LPS-primed and MSU-stimulated system [70]. These findings are mechanistically coherent, but they remain cell-line data and do not demonstrate soluble-urate-driven synovitis in humans.

The framework therefore distinguishes four levels: systemic soluble urate burden; cell-specific urate uptake and export; tissue supersaturation and crystal formation; and context-dependent priming plus crystal-triggered activation. This hierarchy better explains why hyperuricemia is common, why crystal deposition can remain clinically silent, and why acute gout develops only in a subset of individuals and at intermittent time points.

Therapeutically, the goal is not indiscriminate ROS suppression. Physiological ROS participate in signaling and host defense, whereas oxidative stress denotes disrupted redox regulation and/or molecular damage [34,35,36]. Interventions should therefore target defined pathological loops while preserving necessary redox and resolution functions.

## 6. Neutrophils, NETs, Oxidative Burst, and the Paradox of Flare Resolution

Neutrophils dominate the infiltrate during acute gout flares. IL-1β-driven chemokine production recruits neutrophils that phagocytose MSU crystals, generate ROS, release proteases, and form neutrophil extracellular traps (NETs). Human and experimental data indicate that discrete or early NETs can amplify inflammation by promoting macrophage M1 polarization, metabolic reprogramming, and NLRP3 activation; PAD4 deficiency, DNase I, or pharmacological NET inhibition reduced inflammation in experimental models [71]. In a 2025 mouse and cell-culture study, NET-derived material activated the AIM2 inflammasome in synovial fibroblasts and promoted pyroptotic injury; AIM2 knockdown reduced joint inflammation [72]. This expands the potential downstream effects of NETs beyond NLRP3, although human synovial validation is still required.

The transition from amplification to resolution appears to depend on cell density, time, aggregate size, cytokine availability, and clearance. At high neutrophil and crystal density, NETs can coalesce into large NET-MSU aggregates that physically sequester crystals and degrade or limit access to cytokines and chemokines [37]. A 2024 cell-based study further linked expanding NET-MSU aggregates to reduced ERK signaling, increased SHP-1/SOCS-family negative regulators, greater IL-1 receptor antagonist production, and a shift from N1-like to N2-like neutrophil phenotypes [73]. These mechanistic findings are informative but derive partly from differentiated HL-60 cells and require confirmation in primary human synovial neutrophils.

Resolution is therefore not a simple consequence of NET formation itself. It reflects a dynamic balance among early NET-mediated inflammatory signaling, later aggregate formation, macrophage-mediated NET clearance, non-inflammatory crystal processing, and neutrophil fate decisions between NETosis and apoptosis [38,39,40,71,73,74,75,76,77,78,79]. Persistent or poorly cleared NETs may instead sustain tissue injury.

This biphasic biology cautions against broad antioxidant or NET-suppressive treatment without regard to timing. A stage-aware intervention might reduce excessive oxidative burst or pathogenic NET formation during early amplification while preserving aggregate-mediated containment and macrophage-dependent clearance during resolution.

## 7. Lipid Peroxidation and Potential Ferroptotic Mechanisms: Distinct Evidence Levels

Ferroptosis is an iron-dependent regulated cell-death program defined by uncontrolled phospholipid peroxidation when lipid-peroxide repair systems, particularly glutathione peroxidase 4 (GPX4), are insufficient. Core mechanisms include ACSL4-dependent membrane lipid composition, redox-active iron, oxidized phosphatidylethanolamines, and failure of glutathione/GPX4 defenses [80,81,82,83,84,85,86,87]. These criteria are more specific than the presence of ROS or a single oxidative-damage marker.

The first evidence level in gout is measurable lipid peroxidation. Acute inflammation can generate oxidized lipids through mitochondrial stress, neutrophil oxidative burst, cytokine signaling, and tissue injury. Malondialdehyde (MDA), 4-hydroxynonenal (4-HNE), oxidized lipoproteins, and generic lipid ROS indicate oxidative damage, but none is specific for ferroptosis.

The second level is ferroptosis-associated molecular signatures. Urinary-exosome proteomics from patients with acute and intercritical gout identified differential expression of ACSL4, VDAC2, GPX4, and GSS, with exploratory receiver-operating-characteristic performance for acute attacks [88]. Local-lesion proteomics in 2026 reported higher ALOX15 in acute lesions and higher GPX4 and FTH1 in chronic tophaceous lesions [89]. These human observations support stage-dependent perturbation of ferroptosis-related proteins, but they do not demonstrate that cells underwent ferroptotic death.

The third and most stringent level is regulated ferroptotic cell death. Validation would require convergent evidence in human synovium or tophus tissue: compartment-specific lipid peroxidation, altered iron handling, loss of GPX4 function or equivalent repair failure, compatible ultrastructural or molecular features, and functional rescue by ferroptosis-selective interventions such as GPX4 restoration or iron chelation. Bulk-tissue GPX4 expression, MDA, or 4-HNE alone is insufficient.

Recent counter-evidence further supports conservative interpretation. In a 2026 macrophage study, MSU crystals produced lytic death involving overlapping pyroptotic and necroptotic mechanisms; additional inhibition of ferroptosis or ROS did not provide further protection [90]. This does not exclude ferroptosis in other cell types or chronic tissue contexts, but it argues against presenting ferroptosis as a universal mechanism of MSU-induced macrophage death.

Ferroptosis should therefore be treated as a testable, cell-type- and stage-specific hypothesis in gout. Its possible relevance may be greater in persistent synovial or cartilage-adjacent injury than in the earliest IL-1β-driven flare. Future studies should identify the affected cell population, distinguish acute from chronic disease, and include rescue experiments rather than relying on pathway enrichment or single-marker expression.

## 8. Redox-Modulating Therapeutic Strategies: Opportunities and Cautions

The redox-switch framework does not replace urate-lowering therapy. Sustained serum urate reduction remains the cornerstone of disease modification because it prevents crystal formation and promotes crystal dissolution [9,10]. Redox-directed interventions should be evaluated as mechanistically defined adjuncts, not as alternatives to treat-to-target urate control or guideline-based flare treatment.

Allopurinol and febuxostat inhibit XOR and therefore reduce urate generation while also altering an enzymatic ROS source. Their urate-lowering efficacy is clinically established; an additional ROS-specific clinical benefit remains unproven. The 2024 proteomic study of XOI-based treat-to-target therapy identified changes in complement and inflammatory networks but could not separate these effects from urate reduction and changing crystal burden [54].

Colchicine and IL-1 pathway inhibition act primarily on inflammatory and neutrophil pathways rather than as direct antioxidants. They have defined clinical roles in flare treatment, prophylaxis, or selected refractory disease [9,10]. Direct NLRP3 inhibitors remain an active drug-development area, but gout-specific clinical validation is limited [26,27,29,91,92]. A 2026 proof-of-concept study showed that the broad cathepsin inhibitor VBY-825 suppressed crystal-stimulated inflammasome-dependent and inflammasome-independent IL-1β production and reduced experimental gout arthritis, while sparing nigericin- and downstream IL-1-driven responses [93]. This crystal-selective mechanism is promising but remains preclinical.

Nrf2 is a central regulator of antioxidant defense and lipid-peroxide control [94,95]. Its activation has stronger compound-specific preclinical evidence in gout than generic antioxidant language implies. In an MSU ankle model, the Nrf2 activator oltipraz (50–150 mg/kg intraperitoneally) dose-dependently reduced swelling, pain behavior, gait impairment, neutrophil infiltration, ROS, and cytokine production; in macrophages, 25 μM oltipraz improved mitochondrial bioenergetics, and efficacy was lost after Nrf2 inhibition or genetic deletion [96]. These results support pathway dependence in mice but do not establish human efficacy or safe dosing in gout.

Other recent studies illustrate the same translational boundary. Phillyrin reduced oxidative stress and NET formation through KEAP1/NRF2 signaling in experimental gouty arthritis [97]. Astilbin suppressed MSU-induced NET formation through the P2Y6R-IL-8/CXCR2 axis in neutrophil, differentiated HL-60, and mouse experiments [98]. Osteostatin reduced caspase-1 activation, inflammatory mediators, and ROS while increasing nuclear Nrf2 in macrophage and murine models [99]. Defined alginate oligosaccharides activated Nrf2-dependent antioxidant signaling and reduced NLRP3 activation and pain in mice, with loss of benefit in Nrf2-deficient animals [100]. These are mechanistically informative interventions, but none has validated efficacy in human gout.

Earlier studies of gallic acid, curcumin, epicatechin, cichoric acid, and sulforaphane also report effects on Nrf2, NF-κB, NLRP3, ROS, or related pathways [101,102,103,104,105,106,107]. Their interpretation should remain compound-specific. Model choice, route, exposure, formulation, tissue bioavailability, and target engagement differ substantially; positive results cannot be generalized to a class-wide recommendation for “natural antioxidants.”

Vitamin C provides a human translational contrast. Supplementation can modestly lower serum urate in some populations, yet a pilot randomized trial in established gout found a clinically small effect [108,109,110]. Vitamin C should not be presented as a substitute for urate-lowering therapy, and serum urate reduction should not be conflated with evidence of redox-pathway modification within the joint.

**Figure 2:**
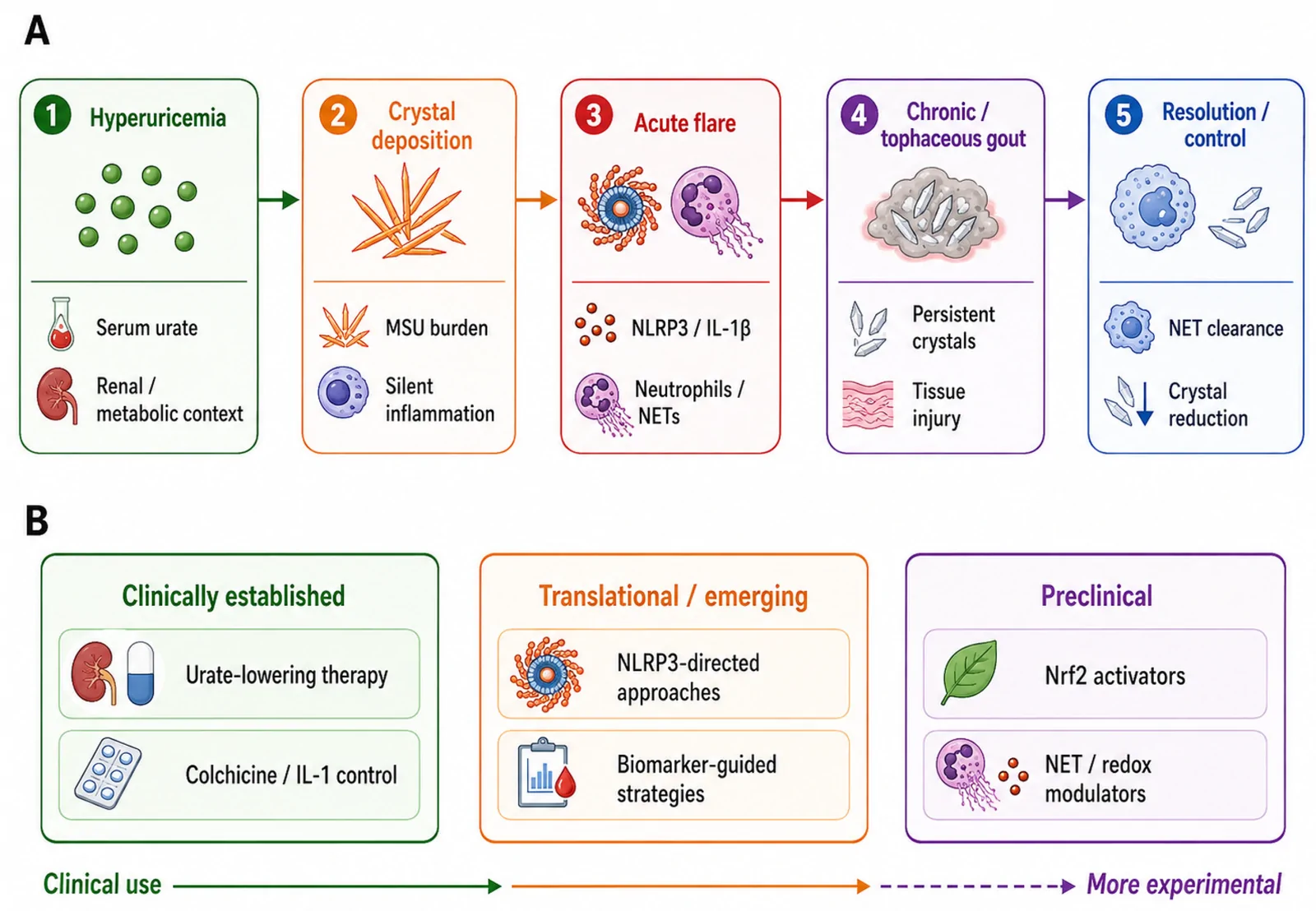
Disease-stage roadmap and therapeutic-strategy maturity in gout. (**A**) The disease-stage roadmap links hyperuricemia, MSU crystal deposition, acute gout flares, chronic/tophaceous gout, and resolution or therapeutic control to distinct inflammatory and redox contexts. The stages represent a conceptual continuum and do not imply that all patients progress sequentially through every phase. (**B**) Therapeutic strategies are grouped according to their highest current level of translational maturity: clinically established approaches, translational or emerging strategies, and predominantly preclinical interventions. Panel (**B**) provides a simplified visual synthesis of Table 2, whereas the table retains compound-specific models, exposures, mechanistic endpoints, and translational limitations. The scheme is intended as a research and evidence-interpretation framework rather than as a validated clinical algorithm.

The stage-specific implications of this framework are summarized in Figure 2A, whereas Figure 2B organizes therapeutic strategies by translational maturity. This combined schematic emphasizes that a redox intervention that is biologically plausible during an acute flare may have a different meaning during asymptomatic hyperuricemia, intercritical disease, chronic tophaceous gout, or resolution, and that mechanistic plausibility does not imply clinical readiness. Recent work on staging, tophus biology, macrophage crystal processing, and neutrophil fate decisions reinforces the need for stage-aware interpretation [21,76,77,79,111].

**Table 2:** Therapeutic implications of the redox-switch model.

Strategy/Compound	Model or Established Use	Evidence Level	Key Translational Limitation
Allopurinol/febuxostat	Treat-to-target urate lowering; XOI proteomic cohorts [54]	Established for urate lowering; mechanistic for ROS/inflammatory networks	ROS-specific benefit cannot be separated from urate and crystal reduction.
Colchicine/IL-1 blockade	Guideline-based flare treatment or selected difficult gout	Clinical	Not direct antioxidant therapy; safety, access, and patient selection remain relevant.
Direct NLRP3 inhibitors	Preclinical and early drug-development programs	Emerging	Limited gout-specific clinical validation.
Oltipraz	MSU ankle mouse model: 50–150 mg/kg i.p.; macrophages: 25 μM [96]	Nrf2-dependent preclinical	No human gout efficacy, exposure, or safety validation.
Phillyrin	Experimental gout; KEAP1/NRF2 and NET endpoints [97]	Preclinical	Formulation, exposure, and human relevance uncertain.
Osteostatin	Macrophage and murine gout models [99]	Preclinical	No clinical pharmacokinetic or comparative efficacy data.
Alginate oligosaccharide AOS3	MSU ankle mouse model; Nrf2-dependence [100]	Preclinical	Natural-product translation and human dosing unresolved.
Vitamin C	Meta-analysis and pilot randomized gout trial [108,109,110]	Human urate-related data	Effect on established gout is small; not a substitute for ULT.
Broad cathepsin inhibition	VBY-825 in crystal-stimulated macrophages and mouse gout arthritis [93]	Preclinical proof of concept	No human exposure, selectivity, safety, or efficacy data.
Astilbin	MSU-stimulated neutrophil/dHL-60 systems and mouse gout model [98]	NET-directed preclinical	P2Y6R pathway and exposure require independent and human validation.

## 9. Biomarkers, Timing, and Patient Stratification

A major unresolved problem in gout is the mismatch between serum urate, crystal load, flare frequency, and inflammatory activity. Serum urate is essential for diagnosis and treat-to-target management but is not a direct marker of joint redox state. A practical biomarker strategy should therefore begin with clinical utility and feasibility rather than with an undifferentiated list of experimental analytes.

Tier 1 markers are clinically available and should anchor all studies: serum urate, creatinine/eGFR, urinary urate or fractional excretion where relevant, C-reactive protein, flare frequency, and imaging-defined crystal burden. They are inexpensive or moderately priced, have same-day to short turnaround, and permit interpretation of any additional redox marker in relation to urate control and disease stage.

Tier 2 translational markers include xanthine/hypoxanthine ratios, XOR activity, IL-1β/IL-18, ASC specks, caspase-1 activity, GSH/GSSG, MDA, 4-HNE, and selected NET complexes such as MPO-DNA. These assays can test mechanistic hypotheses but face pre-analytical instability, assay heterogeneity, limited standardization, and uncertain incremental predictive value. MDA and 4-HNE should be labeled as lipid-peroxidation markers, not as ferroptosis-specific tests.

Tier 3 markers are research-only: urinary-exosome proteins, GPX4/ACSL4/FTH1/ALOX15 panels, targeted lipidomics, metabolomics, spatial transcriptomics, single-cell redox signatures, and tissue imaging. Human urinary-exosome proteomics and lesion proteomics demonstrate feasibility [88,89], while a pilot plasma/urinary metabolomics study identified candidate metabolites distinguishing gout from asymptomatic hyperuricemia [112]. More recent studies show two different stages of biomarker maturity. In a multicenter prospective cohort of 409 patients initiating treat-to-target urate lowering with colchicine prophylaxis, a four-metabolite score was associated with six-month flare outcomes and retained moderate discrimination in a validation cohort [113]. By contrast, a 2026 targeted-metabolomics study distinguishing gout from asymptomatic hyperuricemia involved 47 gout cases and emphasized that external validation is essential [114]. These data justify prospective metabolomic research but do not yet support routine clinical use.

Specimen and timing are critical. Blood is accessible but may not reflect synovial biology; urine is attractive for non-invasive monitoring but is influenced by renal function and concentration; synovial fluid is more proximal to the disease process but invasive; tissue provides spatial specificity but is generally limited to surgery or tophus sampling. Acute flare, intercritical disease, and chronic tophaceous disease should not be pooled without stage-specific analysis.

A feasible worked example would enroll patients beginning urate-lowering therapy and collect Tier 1 measures at baseline and prespecified follow-up visits, with a nested translational substudy obtaining plasma/serum for oxypurines, validated oxidative-damage markers, and NET complexes. Ultrasound or dual-energy computed tomography would quantify crystal burden. Synovial fluid would be collected only when clinically indicated. The primary question would be whether the biomarker panel predicts flare trajectory or crystal resolution beyond serum urate and renal function, not whether any single redox marker differs between groups.

Table 3 prioritizes candidate biomarkers by specimen, accessibility, indicative relative cost, turnaround, analytical specificity, and current validation status. Cost and turnaround categories are approximate and institution-dependent. None of the Tier 2 or Tier 3 markers is ready for routine gout care without prospective validation and assay harmonization.

## 10. Evidence Maturity, Limitations, and Future Research Priorities

Several evidence gaps require explicit separation. First, most mechanistic studies use cell culture or acute MSU models and cannot reproduce chronic human gout, comorbidity, fluctuating urate exposure, tissue adaptation, tophus biology, or long-term urate-lowering therapy. Human synovial, tophus, and longitudinal cohort studies remain limited.

Second, the clinical contribution of XOR-derived ROS cannot currently be separated from urate lowering. Priority studies should combine urate and crystal-burden measurements with oxypurines, tissue or circulating XOR activity, validated redox markers, and clinical outcomes. Designs that compare interventions producing similar urate reduction but different XOR/redox effects would be particularly informative. Parallel tissue-resolved transcriptomic, proteomic, spatial, and activity-based studies should determine whether synovial and tophus XOR biology differs from circulating, hepatic, renal, or vascular compartments.

Third, NLRP3 activation is established, but the patient-level determinants of its threshold remain incompletely defined. Human studies should integrate metabolic priming, renal function, mitochondrial and lysosomal stress, cell-specific signaling, and disease stage rather than assuming a single ROS-dependent pathway.

Fourth, lipid peroxidation and ferroptosis must remain separate research questions. Near-term work should validate lipid-peroxidation assays and spatially identify ferroptosis-related proteins in human samples. Intermediate work should demonstrate iron dysregulation, GPX4 functional failure, and cell-type-specific signatures. Long-term validation requires intervention or rescue evidence showing that selective modulation of ferroptosis changes clinically relevant gout outcomes.

Fifth, Nrf2 activators and natural compounds require pharmacokinetic and translational discipline. Studies should report formulation, exposure, tissue target engagement, comparator treatment, and clinically meaningful endpoints. The most promising compounds should advance only after reproducible pathway-dependent efficacy and safety are established in multiple models.

The framework uses five evidence categories. Established pathways can be stated confidently. Mechanistically supported pathways should be presented as biologically plausible but incompletely translated. Human-associative findings require prospective and tissue-level validation. Conceptual frameworks organize evidence but do not constitute validated biochemical mechanisms or clinical classifications. Emerging or unvalidated findings should remain explicitly hypothesis-generating and should not be converted into therapeutic claims on the basis of pathway enrichment, a single marker, or an animal model.

This hierarchy strengthens interpretive rigor and directly addresses where evidence is sufficient, where uncertainty remains, and which experiment would change the conclusion.

**Figure 3:**
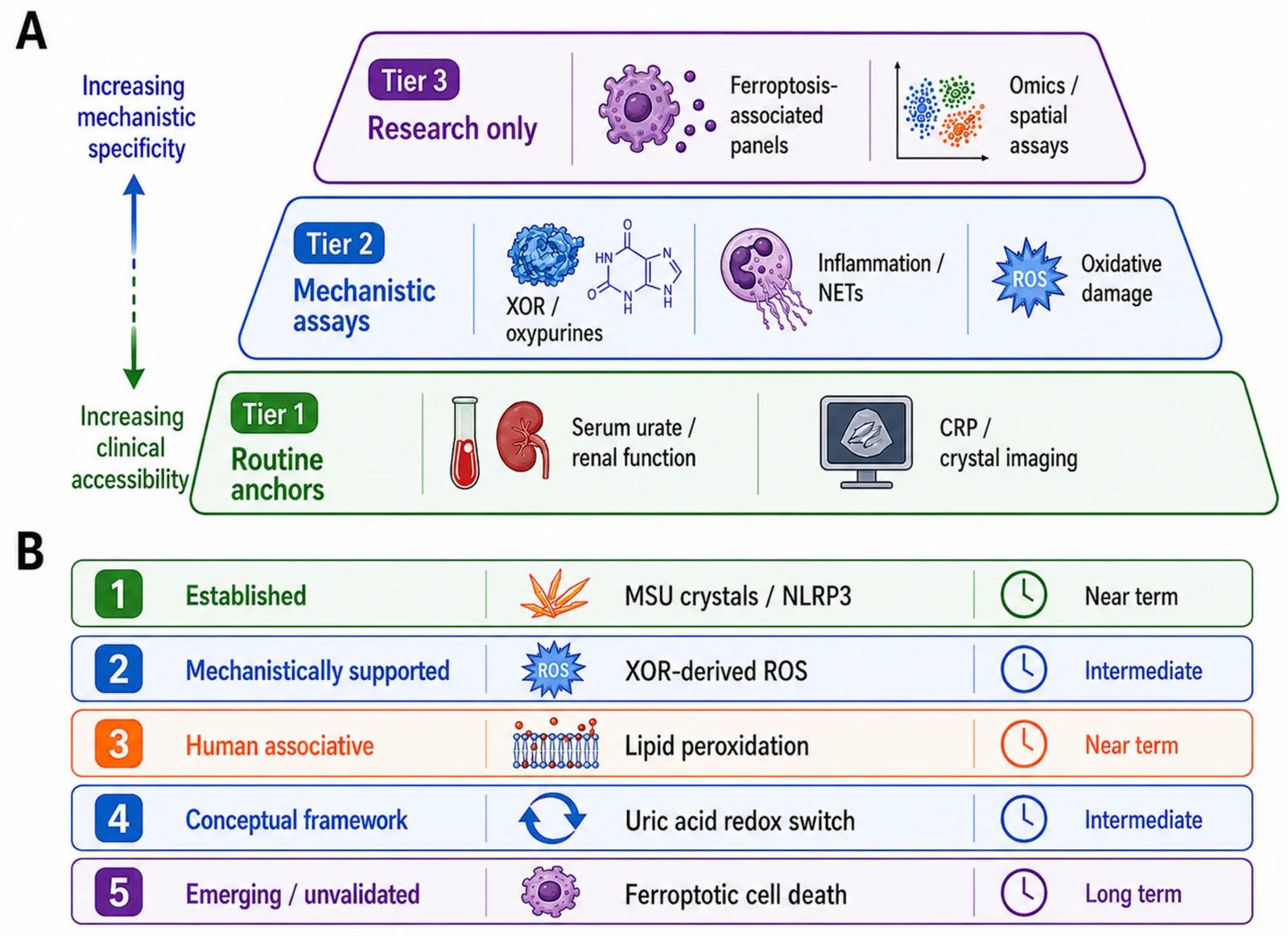
Biomarker prioritization, evidence maturity, and validation horizons in the uric acid redox-switch framework. (**A**) Candidate biomarkers are organized into Tier 1 routine clinical anchors, Tier 2 specialized mechanistic assays, and Tier 3 research-only approaches according to current accessibility, analytical complexity, and mechanistic specificity. Panel (**A**) visually summarizes the prioritization framework presented in Table 3, while the table provides detailed information on specimen type, approximate cost, turnaround time, analytical limitations, and current validation status. (**B**) Major claim domains are classified as established, mechanistically supported, human associative, conceptual, or emerging/unvalidated and are linked to proposed near-term, intermediate, or long-term validation horizons. These horizons are research-planning categories rather than formal predictions. Panel (**B**) summarizes the evidence-maturity and validation framework detailed in Table 4.

Figure 3A translates the practical biomarker tiers from Table 3 into a visual hierarchy of clinical accessibility, whereas Figure 3B integrates the evidence-maturity categories and validation horizons summarized in Table 4.

## 11. Strengths, Limitations, and Interpretive Boundaries

This review has several strengths. It integrates clinical gout biology with purine metabolism, redox chemistry, inflammasome signaling, neutrophil amplification and resolution, lipid peroxidation, and emerging cell-death hypotheses. It also separates evidence by experimental context and adds recent original human and translational studies rather than relying predominantly on secondary reviews.

The principal limitation is the structured narrative design. Scopus and Web of Science Core Collection search strings, date limits, record counts, deduplication, computational organization, and evidence-appraisal logic are reported to improve transparency, and PubMed/MEDLINE was used for targeted verification. Nevertheless, candidate selection was not conducted under a registered systematic-review protocol, no formal risk-of-bias instrument was applied, and inclusion required author judgment. The broad searches also retrieved many records centered on hyperuricemia, natural products, or adjacent metabolic disease, so inclusion in the narrative depended on direct mechanistic or translational relevance to gout.

A second boundary concerns terminology. Oxidative stress, antioxidant capacity, redox signaling, lipid peroxidation, and ferroptosis are not interchangeable. Similarly, molecular associations cannot be assumed to establish causality or therapeutic tractability. This review deliberately uses conservative language where human validation is absent.

The resulting research agenda is practical: integrate serum urate and imaging-defined crystal burden with validated redox and inflammatory markers; isolate urate-dependent from ROS-specific effects of XOR inhibition; define cell type and disease stage in ferroptosis studies; and advance redox-modulating compounds only with pharmacokinetic, target-engagement, and human-relevance data.

## 12. Conclusions

Gout is a crystal-driven inflammatory disease whose clinical heterogeneity is influenced by immunometabolic and redox context. Purine metabolism, XOR-derived ROS, mitochondrial stress, NLRP3 signaling, and neutrophil responses can be integrated without reducing gout to a nonspecific oxidative-stress disorder.

The redox-switch concept is useful as an organizing model because it distinguishes extracellular antioxidant contributions of soluble urate from intracellular, enzymatic, crystal-associated, and inflammatory contexts. It also accommodates the biphasic role of NETs in amplification and resolution. However, urate-independent clinical benefits of XOR-derived ROS suppression remain unproven, and soluble-urate inflammasome findings from primed experimental systems should not be equated with canonical MSU crystal activation.

Human studies now report lipid-peroxidation and ferroptosis-associated protein signatures, but evidence of regulated ferroptotic cell death in gout remains insufficient. Urate-lowering therapy therefore remains the foundation of care. Future redox-directed strategies should be pathway-specific, stage-aware, pharmacokinetically credible, and evaluated with validated biomarkers and clinically meaningful outcomes.

## Figures and Tables

**Table 1:** Redox sources, mediators, and potential gout relevance.

Source/Pathway	Principal Redox Signal	Cellular Context	Interpretation in Gout
Xanthine oxidoreductase	Superoxide; hydrogen peroxide	Liver, endothelium, kidney, macrophages	Dual-output node for urate generation and ROS; ROS-specific clinical effect remains unproven.
Mitochondrial dysfunction	Mitochondrial ROS; oxidized mtDNA	Macrophages, synoviocytes, neutrophils	Can modulate NLRP3 thresholds and metabolic stress; not a single obligatory trigger.
NADPH oxidase/oxidative burst	Superoxide and downstream oxidants	Neutrophils; macrophages	Supports inflammatory amplification and selected NET programs.
MSU crystal uptake	Lysosomal damage; ionic fluxes; ROS	Macrophages, monocytes, synovial cells	Canonical activation context for NLRP3 and IL-1β maturation.
NET biology	ROS, chromatin-protein networks	Acute and resolving joint inflammation	Discrete NETs may amplify; large NET-MSU aggregates may support containment and resolution.
Lipid peroxidation	MDA, 4-HNE, oxidized phospholipids	Synovium, immune cells, cartilage-adjacent tissue	Measurable oxidative injury; not synonymous with ferroptosis.
Systemic oxidative balance	MDA; glutathione; total antioxidant capacity	Circulating blood markers	Human cross-sectional association with inflammation [44]; not joint-specific and not causal.

**Table 3:** Practical prioritization of candidate biomarkers for redox-focused gout studies.

Tier/Domain	Candidate Marker and Specimen	Indicative Access/Relative Cost/Turnaround	Specificity and Analytical Issue	Current Role/Stage
Tier 1: urate and renal context	Serum urate, creatinine/eGFR, urine urate or FEUA	Routine/low/same day	High clinical interpretability; not direct redox measures	Clinical anchor across all stages
Tier 1: inflammation and crystal burden	CRP/ESR; ultrasound or DECT	Routine-to-specialist/low-moderate/same day to days	CRP nonspecific; imaging measures deposits rather than redox state	Flare context and longitudinal response
Tier 2: purine/XOR	Xanthine, hypoxanthine, XOR activity; blood	Specialized/moderate-high/days	Pre-analytical and assay standardization limitations	Translational; especially during XOI studies
Tier 2: oxidative damage	MDA, 4-HNE, protein carbonyls; plasma/serum	Specialized/moderate-high/days	General lipid/protein oxidation; not ferroptosis-specific	Acute flare and chronic injury hypotheses
Tier 2: inflammasome/NETs	IL-1β/IL-18, ASC specks, MPO-DNA; blood or synovial fluid	Specialized/moderate-high/days	Low abundance and variable assays; stage-sensitive	Mechanistic cohorts
Tier 3: ferroptosis-associated	GPX4, ACSL4, FTH1, ALOX15; urine exosomes/tissue	Research-only/high/weeks	Marker association does not prove regulated cell death	Stage-specific validation only
Tier 3: omics/spatial assays	Lipidomics, metabolomics, single-cell or spatial profiling	Research-only/very high/weeks to months	Batch effects, complex analysis, limited scalability	Discovery and cell-type localization
Tier 3: predictive metabolomics	Untargeted or targeted metabolite panels; plasma/serum	Research-only/high-very high/weeks	Model optimism, population specificity, batch effects, external validation required	Prospective flare prediction and gout-versus-asymptomatic-hyperuricemia research [113,114]

**Table 4:** Evidence maturity, interpretive boundaries, priority experiments, and proposed validation horizons.

Claim Domain	Evidence Maturity	Interpretive Boundary	Priority Experiment	Proposed Horizon
MSU crystals/NLRP3	Established	Central IL-1β pathway; upstream control is multi-signal	Human stage-specific threshold determinants	Near term
XOR-derived ROS	Mechanistically supported	Independent clinical benefit beyond urate lowering is unproven	Matched urate lowering with parallel XOR/redox measurements	Intermediate
Lipid peroxidation	Human associative	MDA/4-HNE indicate oxidation, not ferroptosis	Standardized longitudinal assays linked to tissue and outcomes	Near term
Uric acid redox switch	Conceptual framework	Context framework, not a binary biochemical mechanism	Validate markers distinguishing urate burden from redox state	Intermediate
Ferroptotic cell death	Emerging/unvalidated	Pathway proteins alone do not establish regulated death	Human tissue localization plus selective rescue or intervention studies	Long term

## Data Availability

No new data were created or analyzed in this study. All evidence discussed in this review is available in the cited publications. The complete database-search strategy, record counts, deduplication procedure, eligibility criteria, and evidence-appraisal approach are reported in Section 2.

## Abbreviations

The following abbreviations are used in this manuscript:

4-HNE	4-hydroxynonenal
ABCG2	ATP-binding cassette subfamily G member 2
ACSL4	acyl-CoA synthetase long-chain family member 4
AIM2	absent in melanoma 2
ALOX15	arachidonate 15-lipoxygenase
ASC	apoptosis-associated speck-like protein containing a CARD
ATP	adenosine triphosphate
CD38	cluster of differentiation 38
citH3	citrullinated histone H3
CRP	C-reactive protein
CXCR2	C-X-C chemokine receptor 2
DECT	dual-energy computed tomography
dHL-60	differentiated HL-60 cells
eGFR	estimated glomerular filtration rate
ERK	extracellular signal-regulated kinase
ESR	erythrocyte sedimentation rate
FEUA	fractional excretion of uric acid
FTH1	ferritin heavy chain 1
GBD	Global Burden of Disease
GPX4	glutathione peroxidase 4
GSH	reduced glutathione
GSS	glutathione synthetase
GSSG	glutathione disulfide
HO-1	heme oxygenase 1
Hs-CRP	High-sensitivity C-reactive protein
IL-1β	interleukin-1 beta
IL-6	interleukin-6
IL-8	interleukin-8
IL-18	interleukin-18
IRF5	Interferon regulatory factor 5
KEAP1	Kelch-like ECH-associated protein 1
LPS	lipopolysaccharide
MDA	malondialdehyde
MPO	myeloperoxidase
mtDNA	Mitochondrial DNA
MSU	monosodium urate
MyD88	myeloid differentiation primary response 88
NAD+	Oxidized nicotinamide adenine dinucleotide
NETs	neutrophil extracellular traps
NF-κB	nuclear factor kappa B
NLRP3	NOD-like receptor family pyrin domain-containing 3
NOX	NADPH oxidase
Nrf2	nuclear factor erythroid 2-related factor 2
P2X7R	P2X purinoceptor 7
P2Y6R	P2Y purinoceptor 6
PAD4	peptidyl arginine deiminase 4
PBMCs	peripheral blood mononuclear cells
ROS	reactive oxygen species
RIS	Research information systems citation format
SHP-1	Src homology region 2 domain-containing phosphatase 1
SOD	superoxide dismutase
SOCS	suppressor of cytokine signaling
Syk	spleen tyrosine kinase
TNF-α	tumor necrosis factor alpha
THP-1	Human monocytic leukemia cell line
ULT	urate-lowering therapy
URAT1	urate transporter 1 (SLC22A12)
VDAC2	voltage-dependent anion channel 2
XDH	xanthine dehydrogenase
XOI	xanthine oxidase inhibitor
XOR	xanthine oxidoreductase
